# *In silico* and *in vitro* potentials of crocin and amphotericin B on *Leishmania major*: Multiple synergistic mechanisms of actions

**DOI:** 10.1371/journal.pone.0291322

**Published:** 2023-09-08

**Authors:** Ehsan Salarkia, Iraj Sharifi, Alireza Keyhani, Razieh Tavakoli Oliaee, Ahmad Khosravi, Fatemeh Sharifi, Mehdi Bamorovat, Zahra Babaei

**Affiliations:** 1 Leishmaniasis Research Center, Kerman University of Medical Sciences, Kerman, Iran; 2 Basic Sciences in Infectious Diseases Research Center, Shiraz University of Medical Sciences, Shiraz, Iran; 3 Research Center of Tropical and Infectious Diseases Kerman University of Medical Sciences, Kerman, Iran; Iran University of Medical Sciences, ISLAMIC REPUBLIC OF IRAN

## Abstract

A significant barrier to optimal antileishmanial treatment is low efficacy and the emergence of drug resistance. Multiple approaches were used to monitor and assess crocin (a central component of saffron) mixed with amphotericin B (AmpB) potential *in silico* and *in vitro* consequences. The binding behavior of crocin and iNOS was the purpose of molecular docking. The results showed that crocin coupled with AmpB demonstrated a safe combination, extremely antileishmanial, suppressed *Leishmania* arginase absorption, and increased parasite death. This natural flower component is a robust antioxidant, significantly promoting the expression of the Th1-connected cytokines (IL12p40, IFN-γ, and TNF- α), iNOS, and transcription factors (Elk-1, c-Fos, and STAT-1). In comparison, the expression of the Th2-associated phenotypes (IL-10, IL-4, and TGF-β) was significantly reduced. The leishmanicidal effect of this combination was also mediated through programmed cell death (PCD), as confirmed by the manifestation of phosphatidylserine and cell cycle detention at the sub-GO/G1 phase. In conclusion, crocin with AmpB synergistically exerted *in vitro* antileishmanial action, generated nitric oxide and reactive oxygen species, modulated Th1, and Th2 phenotypes and transfer factors, enhanced PCD profile and arrested the cell cycle of *Leishmania major* promastigotes. The main action of crocin and AmpB involved wide-ranging mechanistic insights for conducting other clinical settings as promising drug candidates for cutaneous leishmaniasis. Therefore, this combination could be esteemed as a basis for a potential bioactive component and a logical source for leishmanicidal drug development against CL in future advanced clinical settings.

## Introduction

Leishmaniasis is a neglected and intricate transmittable illness produced by *Leishmania* species and transmitted by female phlebotomine sandflies. It is a zoonotic disease with significant morbidity and mortality, commonly found in humans, rodents, and canines [1–3]. This disease has various clinical presentations ranging from benign or mild symptoms to non-healing chronic form and ultimate death if left without treatment. Visceral leishmaniasis (VL) is the most deadly and systemic form [4], while cutaneous leishmaniasis (CL) is the furthermost type found in urban (dry) and rural (wet) natures universally. World Health Organization has considered the disease one of the six major tropical diseases endemic in 101 countries and territories where approximately 80% of the burden exists in the Eastern Mediterranean Region [5, 6].

During blood feeding, the sandflies inoculate the infective stage or stationary phase promastigotes into the host’s body. Next, macrophages phagocytize metacyclic promastigotes in the skin, transforming them into amastigotes (Leishman bodies). Amastigotes proliferate in phagocytes and affect various tissues, producing different clinical forms of leishmaniasis depending upon tissue specificities. Sandflies become infected when they ingest infected macrophages bearing multiplicated amastigotes. The parasites evolve into promastigotes, proliferate into metacyclic forms, and move to the foregut once they reach the midgut. macrophages are the primary cells where *Leishmania* reproduces and lives for extended durations [7].

A protective immunological response against *Leishmania* species is initiated mainly by Th1 cell phenotypes, which are vital for inducing macrophage oxidative stress machinery, and reactive oxygen species (ROS), resulting in parasite death. In contrast, a primary Th2 response is harmful to the patient. In this situation, arginase (ARG) and inducible nitric oxide synthase (iNOS) signify two possible immune response pathways. While the former is critical for its development, the latter control parasite growth [8, 9].

Pentavalent antimonials namely meglumine antimoniate (MA; Glucantime^®^) and sodium stibogluconate (Pentostam^®^) are the preferred drugs; however, their use is limited due to sporadic lethal adverse effects, parasite resistance, poor treatment adherence, and low efficacy profile [10]. Similarly, the application of alternative drugs including paromomycin, miltefosine, allopurinol, pentamidine, amphotericin B (AmpB), and azole compounds is problematic [10–12].

Presently, leishmaniasis has no effective vaccines, and the available first and second-line treatment choices alone or dual are inadequate. They are associated with severe adverse effects, high cost, long-duration treatment, recrudescence, and *Leishmania* resistance [13]. Other biological control strategies using chemicals are not eco-friendly and unfeasible. Plant-derived products mixed with already available medications have been demonstrated in recent research to be an innovative and synergistic method of treating leishmaniasis. As numerous plant constituents possess immunomodulatory, antimicrobial, and anti-protozoal effects, within normal limits toxicity, they can be considered a substitute medicine source for treating communicable diseases in endemic countries [14]. Certainly, several steps including safety, efficacy, and standardization are essential before being registered and marketed. Furthermore, elucidating the mode of action of natural constituents can significantly advance drug development strategies.

*Crocus sativus L*. is a herbaceous flowering plant in the Iridaceae family that blooms in the fall, contains the red stigma (or saffron), and is extensively cultured in Iran and to a minor extent in Greece, India, Spain, Morocco, Greece, Italy, France, Azerbaijan, and China, [15–17]. Over 150 active components from saffron have been isolated and available for commercial and medical applications. Antioxidant and anti-inflammatory properties of saffron have also been studied and confirmed for use as drugs that combat depression, malignancy, and other cardiovascular and neurodegenerative conditions [18, 19]. In traditional medicine, saffron promotes blood circulation, removes blood stasis, and relieves [20]. Polyphenols comprising flavonoids abundant in saffron include hesperidin, quercetin, rutin, luteolin, and bioflavonoids. The three critical components of saffron are crocin, which gives the yellow pigment from the stigmas; picrocrocin, which accounts for the corroded, bittersweet taste; and safranal lends the earthy smell to the spice. Crocin is one of the essential alkaloid ingredients of saffron and has many beneficial effects on health. Anticancer, antioxidant, memory, and learning skills properties and increased blood flow in choroid and retina, antimicrobial, and antidiabetic have been demonstrated in many studies [21–23].

AmpB is a polyene macrolide used to treat leishmaniasis among various conventional formulations. The discerning activity of this antibiotic against *Leishmania* species is due to its superior affinity targeting ergosterols biosynthesis, which is more principal in the plasma membrane of these parasites than the cholesterol existing in the mammalian cell membranes [24]. Combining different drugs with various mechanistic actions can provide potential synergism, hence exerting a more significant therapeutic effect in treating disease, and this signifies the most promising approach for developing novel leishmanicidal preparations [25].

The previous investigation revealed that crocin possesses an inhibitory effect on the stages of *Leishmania major* [26]. This study aimed to explore crocin and AmpB on *L*. *major* stages as a model drug employing a broad panel of *in silico-*based and preclinical experimental assays. We precisely targeted multiple *in vitro* approaches to monitor inclusive mechanisms of actions, including molecular modeling, leishmanicidal impacts, safety index, arginase activity, antioxidative and apoptotic values, gene expression fingerprints, and cell cycle profiling.

## Material and methods

### A) Molecular docking

#### Estimate of practical residues of iNOS protein

To detect the hot spot of valuable residues in the construction of iNOS as it the importance of controlling leishmaniasis parasites [27] before tying up, the “Hotspot” (https://loschmidtchemi.muni.cz/hotspotwizard/) and CASTp (http://sts.bioe.uic.edu/castp/index.html?1ycs) software were used [28, 29].

#### Study of physical pockets of iNOS protein area

Measurement of open superficial extents on 3-dimensional (3-D) constructions is essential in advanced experimentations. Molegro Virtual Docker software (Molegro 2011) detected pockets on surfaces and cavities.

#### Protein-ligand docking

The 3-D structure of crocin was achieved by PubChem CID 936 [PubChem https://pubchem.ncbi.nlm.nih.gov/compound/Nicotinamide]. The 3-D form of iNOS was obtained from the Protein Data Bank (PDB) (https://doi.org/10.2210/pdb1hig/pdb). Molecular docking investigations were completed in Molegro ApS (Aarhus 2.5.0, Denmark).

### B) *In vitro* assay

#### Drug preparation

Crocin as the experimental group was purchased from Sigma-Aldrich^®^, USA (Catalog No 42553-65-1). AmpB as a positive control group was purchased from Abbott Company (Abbott India Ltd.). Drugs were dissolved in hot sterile water using serial concentrations of 6.25, 12.5, 25, 50, 100, and 200 μM for testing. In combination treatment, we used a mixture of crocin and AmpB including 6.25+6.25, 12.5+12.5, 25+25, 50+50, 100+100, and 200+200 μM.

#### Antioxidant activity assessment

To assess the antioxidant properties of crocin, the drug was combined with butylated hydroxyanisole (BHA) in a microtube and 2.6 mL of α, α-diphenyl-β-picrylhydrazyl (DPPH) was included in the mixture. The absorbance (optical density; OD) was then calculated with a spectrophotometer at 518 nm. To determine the essential scavenging action, the absorbance values of the samples were measured and used to calculate the percentage of inhibition [30].

#### Parasite and macrophage cultivation

The murine macrophage J774-A1 cell line and the standard *L*. *major* strain (MRHO/IR/75/ER) in stationary phase were attained from the Kerman Leishmaniasis Research Center in Iran. The culture medium of parasites was RPM 1640 and the macrophage cells were DMEM. All media were enriched with 10% FBS and 1% penicillin G and streptomycin. The study was approved by the Medical Ethics Committee of Kerman University of Medical Sciences. The Ethic approval Code is IR.KMU.REC.1396.2155.

#### Anti-promastigote assay

To assess the impact of crocin, AmpB, and their combination on *L*. *major* promastigotes, an anti-promastigote assay was performed. 10^6^ cells of *L*. *major* promastigotes per mL were counted and cultured on a 96-well plate and treated with 20 μl of several concentrations (0, 12.5, 25, 50, and 100 μM) of drugs. Each concentration was repeated in triplicate. Plates were kept at 25±1°C for 72 h, then 5 mg/mL of 3- (4,5-dimethylthiazol- 2‑yl) -2, 5‑diphenyl‑tetrazolium bromide (MTT) was put in each well and incubation continued for 3 h, centrifuged at 3000 rpm for 8 min, and Dimethyl sulfoxide (DMSO) replaced with content, and the OD was measured at 490 nm using a Multi-Mode ELISA (ELX-800-BioTek). The 50% inhibitory concentration (IC_50_) rate was determined using the SPSS package.

#### Anti-amastigote assay

To evaluate the effect of crocin, AmpB, and their combination on *L*. *major* amastigotes, 1×10^5^/mL of J774 murine macrophages in cultured in 6-chamber slides (Lab-Tek, Nalge Nunc NY, USA) and kept at 37±1°C in 5% CO_2_ for 12 h, then the macrophages infected with 1×10^6^/mL of the metacyclic form of promastigotes and incubated for the next 24h (macrophages to parasite ratio at 1:10). After incubation, free parasites and old medium were removed and changed with new fresh medium and 50 μL of 12.5, 25, 50,100 μM of crocin, AmpB, and combination were added to macrophages. After 72 h we used Giemsa stain to prepare the slide counting the amastigotes under a light microscope [31].

#### Cytotoxic effects

To assess the potential cytotoxic effects of crocin, AmpB, or their combination, on the J774 murine macrophages, 5×10^5^ cells/mL were counted and grown with varying concentrations (0–100 μM) of drugs in 96-well plates. Macrophages were incubated for 72 h at 37±1°C with 5% CO_2_. The untreated for 72 h to evaluate the cytotoxic activities of crocin, AmpB, or in combination. The well included culture and macrophages without medicines as an untreated control group. After incubation, a similar amount of the MTT solution as the anti-promastigote assay was used and incubated for an additional 3 h. The OD was read similarly by the ELISA at 490 nm following adding DMSO solution. SPSS software and a probit test were used to calculate the cytotoxic activity at 50% (CC_50_ value). The safety of the drugs was evaluated using the selectivity index (SI), by the following equation (SI = IC50/CC50 ≥ [32]. The combination index (CI) was calculated to evaluate the potential synergy of crocin and AmpB. The formula used for CI calculation was as follows: CI = (D)/(Dx)_i_+(D)/(Dx)_ii_, where (Dx)_i_ and (Dx)ii show the concentrations of crocin and AmpB and (D) represents a combination of crocin and AmpB. The CI value was used to quantitatively define the degree of synergism (CI<1), additive outcome (CI = 1), or antagonism (CI>1) among the two medications. The theoretic IC_50_ was also determined to assess the synergistic activity of the combination therapy. The theoretic IC_50_ was calculated using the following formula: theoretic IC_50_ = (IC_50_ AmpB/2) + (IC_50_ crocin/2).

#### Assessment of arginase level

The activity of arginase in intra-macrophage *L*. *major* amastigotes was measured according to the supplier’s protocol (Sigma-Aldrich^®^, USA, cat. No. MAK112). Several concentrations of crocin, AmpB, or a combination of both were used to treat the infected macrophages. For this measurement, 1×10^6^ intra-cellular amastigotes were lysed with Tris-HCl (10 mM, pH 7.4) and Triton X-100 (0.4%). After that10 mM MnCl_2_ was added to the mixture and maintained at 56°C for 10 min. After centrifugation, 40 μL of supernatant was mixed with 5X substrate buffer and kept for 3h at 37°C. Finally, 200 μL of urea was used to stop the enzymatic reaction at 25±1°C. The arginase activity was computed at 430 nm employing an ELISA reader as follows:

$$Activity=\frac{A\left(\;sample\right)-A\;\left(blank\right)}{A\;\left(standard\right)-A\;\left(water\right)}\times \frac{\left(1mM\times 50\times 10^{3}\;\right)}{\left(V\times T\right)}$$

T = response period

V = sample volume

#### Gene expression analysis

The comparative expression levels of genes were determined using quantitative polymerase chain reaction (qPCR). RNA was removed from harvested cells using a Qiagen RNeasy mini kit was used to extract RNA, after determining its concentration using a NanoDrop spectrophotometer, a TaKaRa cDNA kit was used and qPCR was performed using an SYBR Green experiment in the Corbett Rotorgene 3000 cycler system. The primers and control gene sequences are given in Table 1 [33]. The expression of target genes was evaluated using the 2^-ΔΔCT^ method, and the ΔCT was calculated using the following formula: [ΔCT = CT(target)-CT(control)].

**Table 1:** The specific primers and reference gene sequences.

Template	Forward and reverse sequences (5´-3´)		Product size (bp)
IFN-γ	Forward	5-GCCGATGATCTCTCTCAAGTGAT-3	106
	Reverse	5-ACAGCAAGGCGAAAAAGGATG-3	
IL-12p40	Forward	TGGTTTGCCATCGTTTTGCTG	171
	Reverse	ACAGGTGAGGTTCACTGTTTCT	
TNF-α	Forward	CAGGCGGTGCCTATGTCTC	161
	Reverse	CGATCACCCCGAAGTTCAGTAG	
iNOS	Forward	ACATCGACCCGTCCACAGTAT	89
	Reverse	CAGAGGGGTAGGCTTGTCTC	
Stat-1	Forward	GCTGCCTATGATGTCTCGTTT	154
	Reverse	TGCTTTTCCGTATGTTGTGCT	
c-Fos	Forward	CGGGTTTCAACGCCGACTA	94
	Reverse	TGGCACTAGAGACGGACAGAT	
Elk-1	Forward	TTGTGTCCTACCCAGAGGTTG	168
	Reverse	GCTATGGCCGAGGTTACAGA	
IL-10	Forward	CTTACTGACTGGCATGAGGATCA	134
	Reverse	GCAGCTCTAGGAGCATGTGC	
IL-4	Forward	GGTCTCAACCCCCAGCTAGT	101
	Reverse	GCCGATGATCTCTCTCAAGTGAT	
TGF-β	Forward	CCACCTGCAAGACCATCGAC	112
	Reverse	CTGGCGAGCCTTAGTTTGGAC	
GAPDH	Forward	5-AGGTCGGTGTGAACGGATTTG-3	95
	Reverse	5-GGGGTCGTTGATGGCAACA-3	

#### Determination of NO generation

We use the Griess reaction assay to determine secreted nitric oxide amounts in intra-macrophages amastigotes treated with several concentrations (0–200 μM) of crocin, AmpB, and co-administration. At first J774 murine macrophages (10^5^ cells/mL) were infected by *L*. *major* parasite (10^6^ cells/mL) (macrophage to parasite ratio at 1:10). Cells were kept overnight at 37 °C and 5% CO_2_, after that the culture was refreshed and 10 μL of drug added to each well and incubates for 72 h. After incubation, suspensions in each well were collected and NO release was determined colorimetrically in infected macrophages by the Griess reaction. Briefly, supernatants were composed with LPS, and 100 μL of liquid were incubated with an identical size of Griess reagent (Sigma-Aldrich^®^, USA) (1% sulfanilamide, 0.1% naphthyl ethylenediamine, and 2.5% H3PO4) were added into wells kept for 0 min. An ELISA reader then measured the absorbance at 540 nm (Bio Tek-ELX800) [33].

#### Assessment of reactive oxygen species (ROS)

*L*. *major* intra-cellular amastigotes treated with crocin, AmpB, and combination. After 24 h, the cells were washed away with PBS (pH 7.4) and overloaded with 10 μM of a permeate probe diacetate 2′.7′-dichlorofluorescein (Sigma-Aldrich^®^, USA) was diluted in DMSO and incubated at 37±1°C in a 5% CO2 for 25 min. ROS was measured with the flow cytometer (BD Biosciences)

#### Cell cycle examination

Promastigotes were treated with serial concentrations of crocin, AmpB, or co-administration and incubated at 25±1°C for 72 h. Then, samples were collected in 1 mL of PBS, fixed with absolute methanol, centrifuged, resuspended at 50 μl of RNase (1 mg/mL), and kept in RT for 30 min after that 1 mL of 0.1 mg/mL of propidium iodide. The DNA content was examined by flow cytometry (Becton Dickinson), and the Cell Quest software assessed the proportion of organisms in different cell phases.

#### Annexin V/PI and flow cytometry

The assay was undertaken to detect apoptosis of *L*. *major* promastigotes treated with different concentrations of crocin, AmpB, or combined using PE Annexin V Apoptosis Detection Kit I (BD Pharnigen ^™^). Therefore, 1×10^6^ promastigotes were cultured in a 1.5 mL microtube. Then, 100 μl of various drug concentrations were incubated at 25±1°C for 72 h. After that, the organisms were washed with PBS and maintained in a 1mL binding buffer. Then 100 μl solutions were transferred to a 5-mL tube and 5 μl 7-AAD stains plus 5 μl Annexin V was added to each tube and allowed in the dark at 25±1°C for 15 min. Subsequently, samples were evaluated in a flow cytometer.

### Statistical analysis

Analyses were performed by SPSS v. 22.00 (Chicago, IL, USA) and GraphPad Prism v. 8.0 (CA, USA). One-way ANOVA was used to compare the statistical difference among concentrations of drug and paired t-test was completed to find any statistical difference groups. *P* < 0.05 was set as a significant level. The data of one-way ANOVA test shows in S1 Dataset.

## Results

### Evaluation of the competence of crocin for binding to iNOS

Lys14, Met135, and Leu136 were predicted as suitable amino acids in mutable residues in catalytic pockets and access tunnels.

### Predicting structural protein area

**Fig 1:**
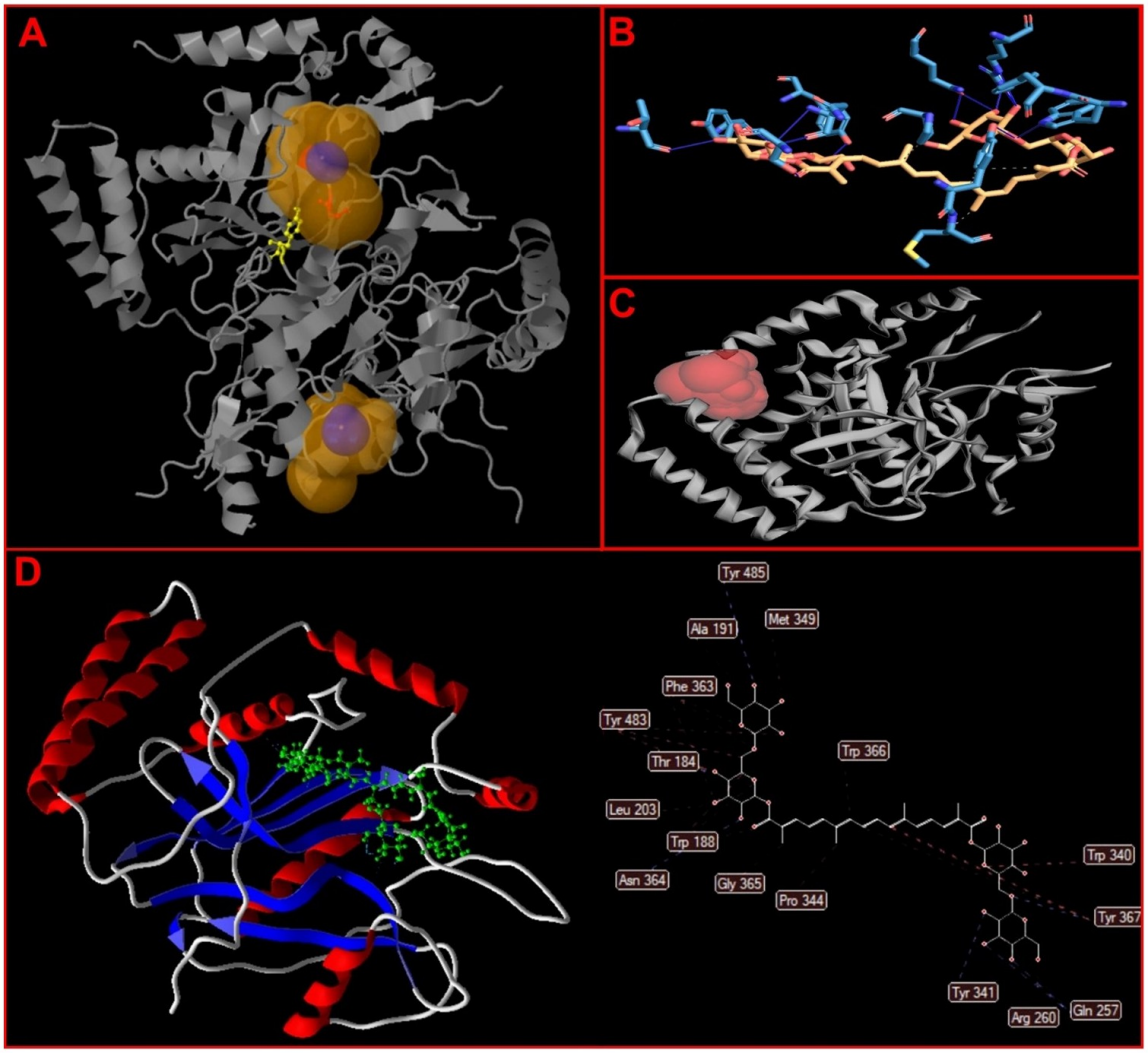
Docking. A) Nitric oxide (NO) consists of a central pocket and 4 cavities. B) Predicted amino acids in pocket formation by PLIP web tool. C) Crocin binds to NO with the active site residues by LIGPLOT program. D) Molecular docking by Molgro Virtual Docker software.

The useful activity of the iNOS protein surface is presented in Fig 1A. We evaluated the 2-D interaction diagrams showing hydrophobic interaction and hydrogen bond necessary energy of the ligand and target protein. We use Ligand Interaction Profiler (PLIP) online server to evaluate the interaction between iNOS and crocin metabolites. Regarding steric relations, crocin interacts with Ser133, Gln134, and Lys13 amino acids of iNOS, respectively (Fig 1B).

Our molecular docking outcomes show crocin binds to iNOS (Fig 1C), and amino acids active site THR, SER, GLN, GLN, ARG, TRP, TYR, and ASN are active site residues (Table 2 and Fig 1D). The results show that the MolDock score was -241.053 kcal/mol.

**Table 2:** Contribution of the iNOS residues/molecules.

**Hydrophobic Interaction**							
Index	Residue		AA	Distance	Ligand atom		Protein atom
1	344A		PRO	3.35	2993		2167
2	367A		TYR	3.58	2986		2362
3	367A		TYR	2.54	2998		2363
4	368A		MET	3.25	2996		2375
**Hydrogen Bonds**							
Index	Residue	AA	Distance H-A	Distance D-A	Donor angle	Donor atom	Acceptor atom
1	184A	THR	2.57	3.05	109.86	29.39 [O3]	690 [O2]
2	236A	SER	3.47	3.79	101.48	1236 [O3]	2955 [O2]
3	257A	GLN	2.21	3.11	152.01	2948 [O3]	1456 [O2]
4	257A	GLN	2.97	3.41	108.38	2944 [O3]	1456 [O2]
5	260A	ARG	3.10	3.97	149.24	1486 [Ng+]	2942 [O3]
6	260A	ARG	2.24	2.68	104.99	1489 [Ng+]	2944 [O3]
7	340A	TRP	3.24	3.78	116.06	2125 [Nar]	2938 [O3]
8	341A	TYR	1.92	2.86	158.70	2942 [O3]	2144 [O3]
9	364A	ASN	3.31	3.94	123.17	2325 [Nam]	2949 [O3]
10	364A	ASN	1.58	2.48	150.84	2949 [O3]	2328 [O2]
11	367A	TYR	2.24	3.19	168.17	2368 [O3]	2938 [O3]
12	483A	TYR	2.87	3.53	126.34	2943 [O3]	22756 [O2]
13	485A	TYR	3.02	3.36	102.01	2792 [O3]	2948 [O3]
14	485A	TYR	2.43	3.36	157.12	2947 [O3]	2792 [O3]

### Effect of crocin on antioxidant action

**Fig 2:**
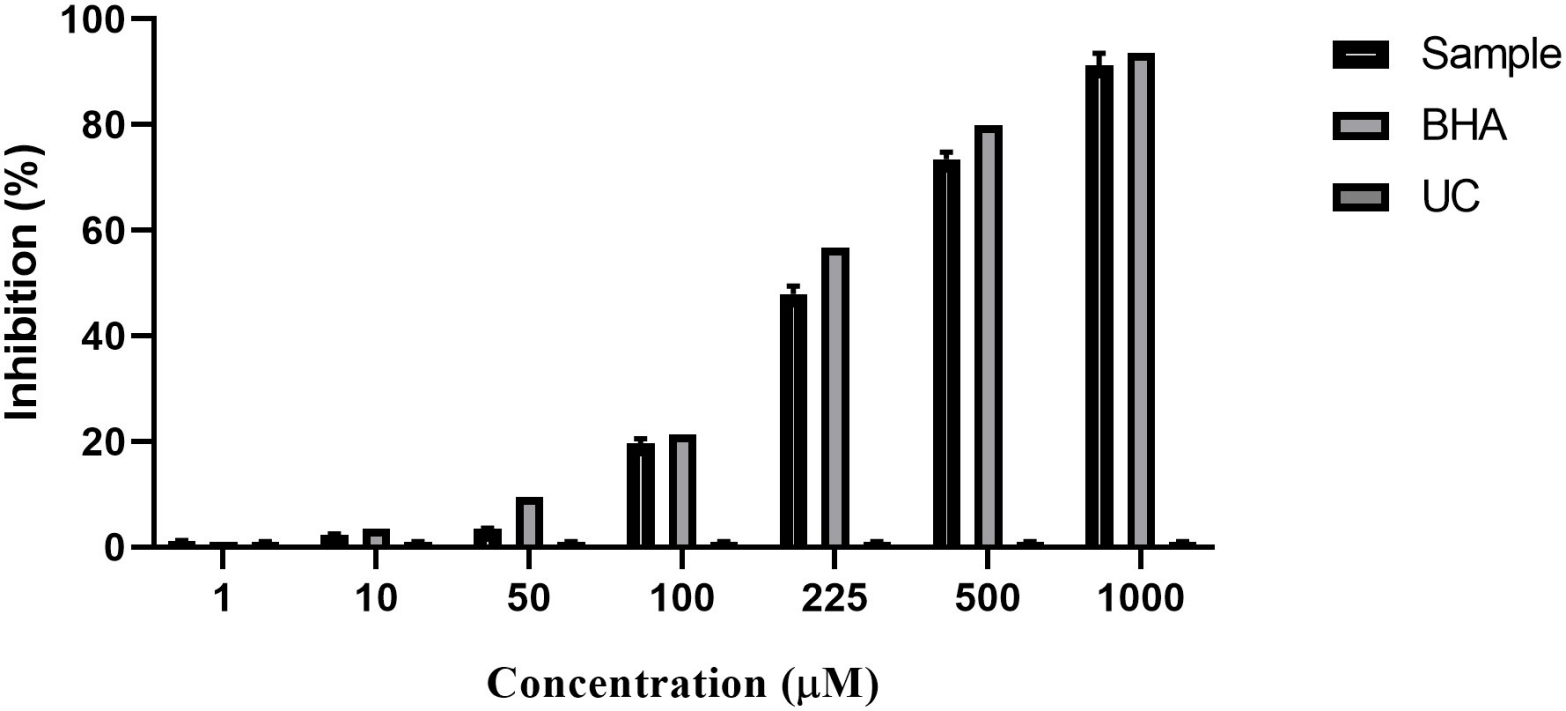
Scavenging effects of crocin on 1, 1-diphenyl-2-picrylhydrazyl (DPPH) free radicals compared to butylated hydroxyanisole (BHA) as a typical control. Data are means ± SD of triplicate tests (There was no significant difference between crocin and BHA).

The compounds ’ hydrogen donations evaluated the fundamental scavenging activity of crocin and BHA on DPPH (Fig 2). The effect was a concentration-effect outcome. There was no significant difference between the crocin antioxidant action and BHA’s. It shows that crocin has potent antioxidant activity.

### Effect of crocin, AmpB, or combination on promastigotes mortality

**Fig 3:**
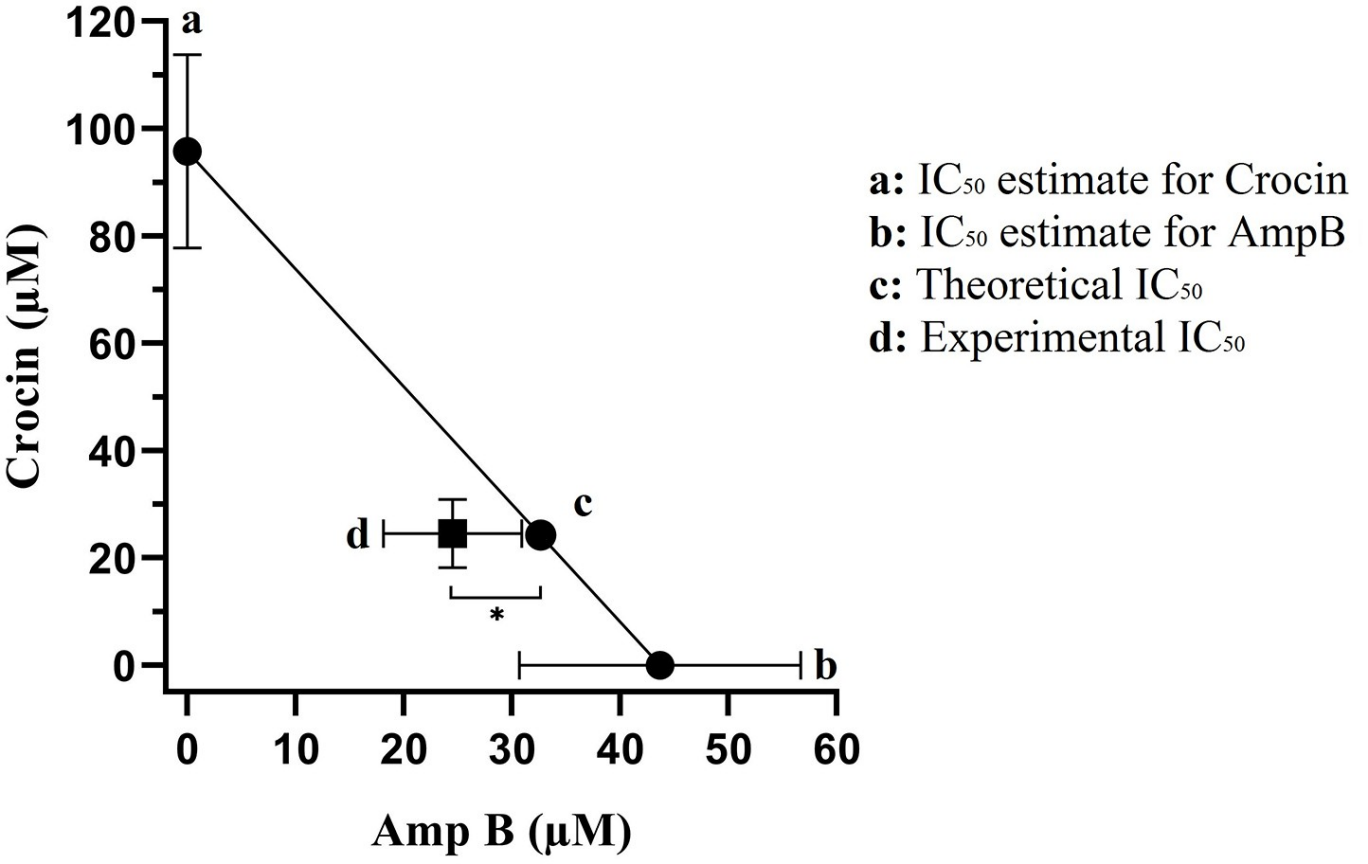
The isobologram analysis of the effects of drugs combination of crocin and AmpB. Foci a and b displayed the IC_50_ value of crocin (95.8 μM) and AmpB (43.7 μM), respectively. Theoretical IC_50_ was 69.75 μM and our experimental IC_50_ was 24.5 μM. Statistical analysis revealed that there was a significant difference between experimental IC_50_ and theoretical IC_50_ (*P<* 0.001).

The mortality profile of treated *L*. *major* promastigotes is accessible in Fig 3. The CC_50_ results showed the superior effect of crocin coupled with AmpB (*P* < 0.0001) on promastigotes than crocin or AmpB (*P* < 0.001) alone relative to the untreated group.

On murine cell lines and DMEM culture media, various drug concentrations (0–200 μM) were applied, and the CC_50_ rates of drugs were calculated using the counting means of intracellular amastigotes. Examination of cytotoxicity at expected drug concentrations indicated that the drugs did not have a lethal effect, as the safety index (SI), which is calculated as CC50/IC50, was found to be within acceptable limits, with an SI value of at least 1. The selectivity index (SI) for AmpB, crocin, and crocin plus AmpB was 7.5, 20.2, and 18.1, respectively (Table 3).

**Table 3:** Evaluating the IC_50_ values of crocin and crocin plus amphotericin B (AmpB) against amastigotes and promastigotes forms of *L*. *major* compared with AmpB and CC_50_ values of the drugs on macrophages using the SI index.

Drugs	Amastigote		Promastigote		Macrophage	^c^SI
	^a^IC_50_ ± SD (μM)	P-value	^a^IC_50_ ± SD (μM)	P-value	^b^CC_50_ (μM)	(Selectivity Index)
**AmpB**	43.7±13	NR	293.5±71	NR	328.7	7.5
**Crocin**	95.8±18	*P* < 0.01	382.7±23	*P* < 0.01	1935.3	20.2
**Crocin + AmpB**	24.5±6.4	*P* < 0.01	229.6±6.2	*P* < 0.01	443.3	18.1

^a^IC_50;_ Drug concentration inhibited 50% of promastigotes and amastigotes growth.

^b^CC_50;_ Drug concentration that inhibited 50% of macrophage growth.

^c^ SI; Selectivity index (CC_50_/IC_50_).

NR: Not related.

### Effect of crocin, AmpB, and combination on amastigotes

The study observed significant differences in the indices of *L*. *major* intra-cellular amastigotes when compared to the negative group (P < 0.001). Table 3 provides the values for IC50, CC50, and the selectivity index (SI) of crocin, AmpB, and the combination of both. We determined that the CI index was 0.81 which is defined as synergism (CI<1) and our analysis verified that theoretical IC_50_ was 69.75 μM had a significant difference from experimental IC_50_ was 24.5 μM (*P<* 0.001) that represented a synergistic effect in our combination activity (Fig 3).

The number of amastigotes was substantially reduced at different concentrations (*P* < 0.001) relative to the control group. Except at 6.25 μM, crocin showed no effect on the average number of amastigotes, while AmpB displayed an extensive decline at different concentrations (*P* < 0.001). In the same situation, however, AmpB was more efficient than crocin at 200 and 300 μM concentration in decreasing the amastigote load (21.3±0.7 vs. 24.4±0.6, respectively).

Each drug alone was not entirely effective as some organisms were still alive. The IC_50_ values for the combination of crocin and AmpB, on intracellular amastigotes, were significantly lesser (24.5±6.4 μM) than crocin (95.8±18 μM) or AmpB (43.7±13μM) (*P* < 0.01). Compared to the amastigotes, crocin, AmpB, or combinations, they represented higher IC_50_ values on promastigotes (382.7±23, 293.5±71, 229.6±6.2 respectively).

The activity of crocin and AmpB alone were comparable; however, at 6.25 μM, crocin did not exhibit any response in killing the intracellular amastigotes (Table 4). However, the crocin/AmpB mixture effect was enhanced, and at a 200 μM combination, no amastigotes were alive (*P* < 0.001) (Table 5).

**Table 4:** The effect of different concentrations of crocin and amphotericin B (AmpB) on the mean number of intra-macrophage amastigotes.

Concentration (μM)	Crocin		AmpB	
	Mean ± SD	P value	Mean ± SD	P value
**0.0 (Control)**	39.7±3.7	NR	39.7±3.6	NR
**6.25**	35.8±2.4	*P* > 0.05	28.2±0.6	*P* < 0.001
**12.5**	32.3±1.4	*P* < 0.01	27.4±0.07	*P* < 0.001
**25**	27.1±1.4	*P* < 0.001	24.3 ±0.7	*P* < 0.001
**50**	23.8±0.3	*P* < 0.001	22±1.0	*P* < 0.001
**100**	22.6±0.5	*P* < 0.001	16.1±0.2	*P* < 0.001
**200**	13.25±1.5	*P* < 0.001	7.32±2.4	*P* < 0.001

**Table 5:** The effect of different concentrations of crocin plus amphotericin B (AmpB) on the mean number of intra-macrophage amastigotes.

Concentration (μM)	Crocin + AmpB	
	Mean ± SD	P-value
0.0 (Control)	39.7±3.6	NR
6.25+6.25	26.8±0.02	*P* < 0.001
12.5+12.5	26.0±0.5	*P* < 0.001
25+25	20.8±1.0	*P* < 0.001
50+50	16.6±0.6	*P* < 0.001
100+100	13.5±0.4	*P* < 0.001
200+200	0±0	*P* < 0.001

**Fig 4:**
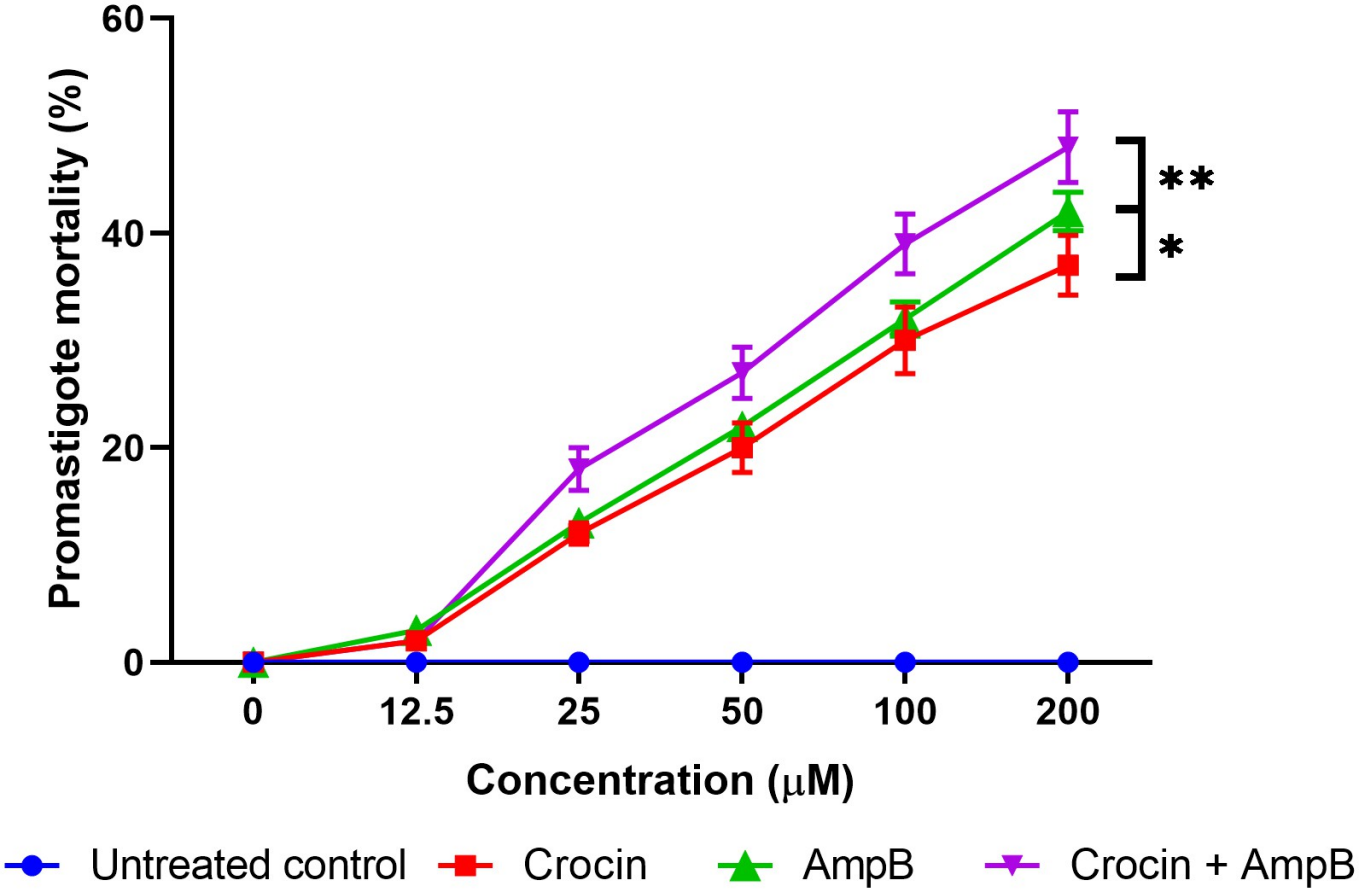
The mortality rate of *Leishmania major* promastigotes at different crocin and amphotericin B (AmpB) concentrations, alone or combined relative to untreated control by MTT assay. (*P < 0.001), **(*P* < 0.0001).

Fig 4 exhibits the mean mortality rates of *L*. *major* promastigotes (nonclinical stage) treated with various concentrations of crocin, AmpB, or both. The results demonstrated that crocin and AmpB alone were meaningfully effective (*P* < 0.001) against *L*. *major* promastigotes, but their action was significantly improved (*P* < 0.0001) in combination.

### Effect of crocin, AmpB, or combination on arginase activity

**Fig 5:**
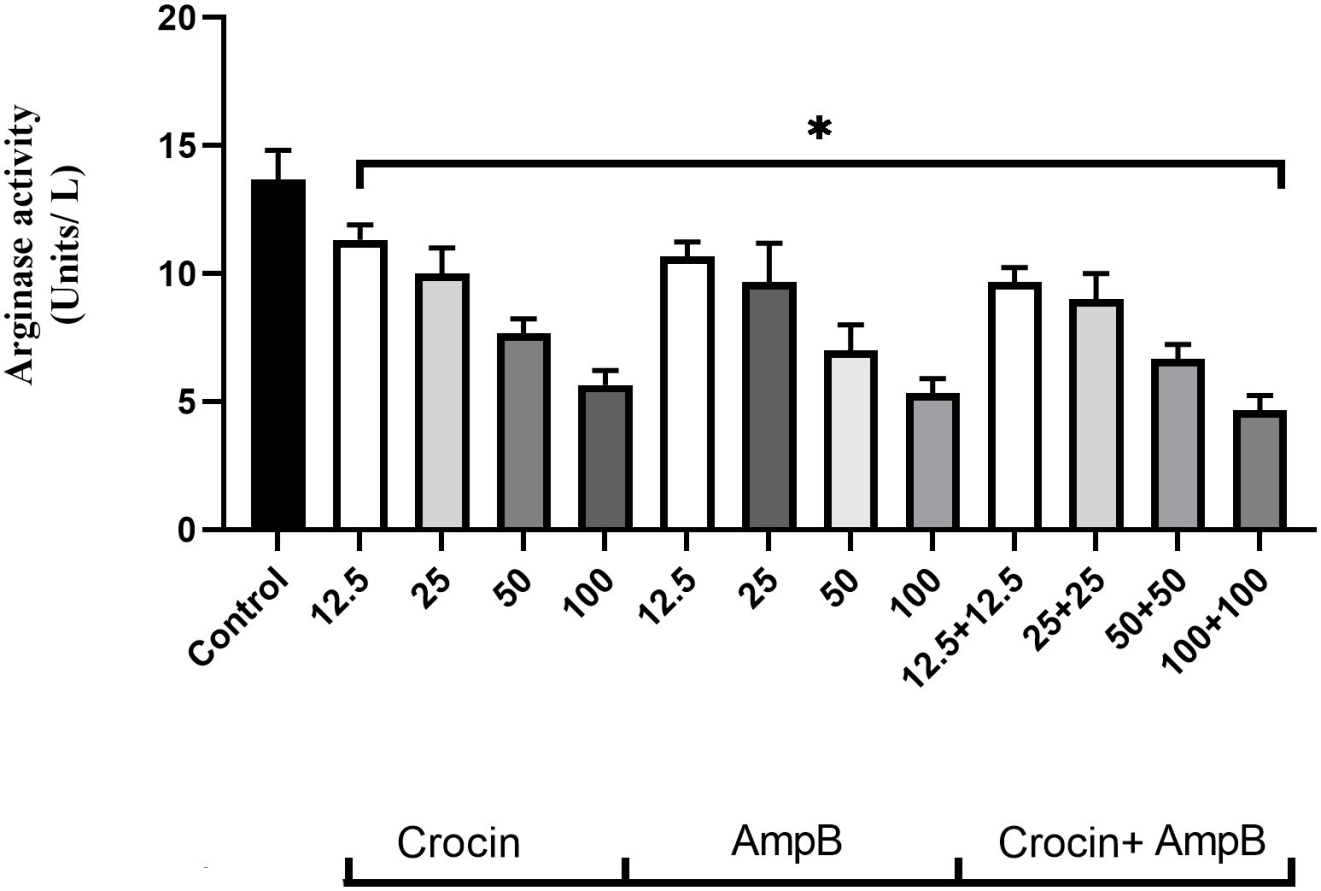
Effect of crocin, amphotericin B (AmpB), and combination on arginase activity levels of treated macrophages (*P < 0.001 compared to the untreated control group).

The outcome presented that by increasing the concentration of crocin, AmpB, and a combination of them, ARG activity levels of treated macrophages significantly decreased (*P* < 0.001) relative to the untreated control group (Fig 5).

### Effect of crocin, AmpB, and combination on the gene expression profile

**Fig 6:**
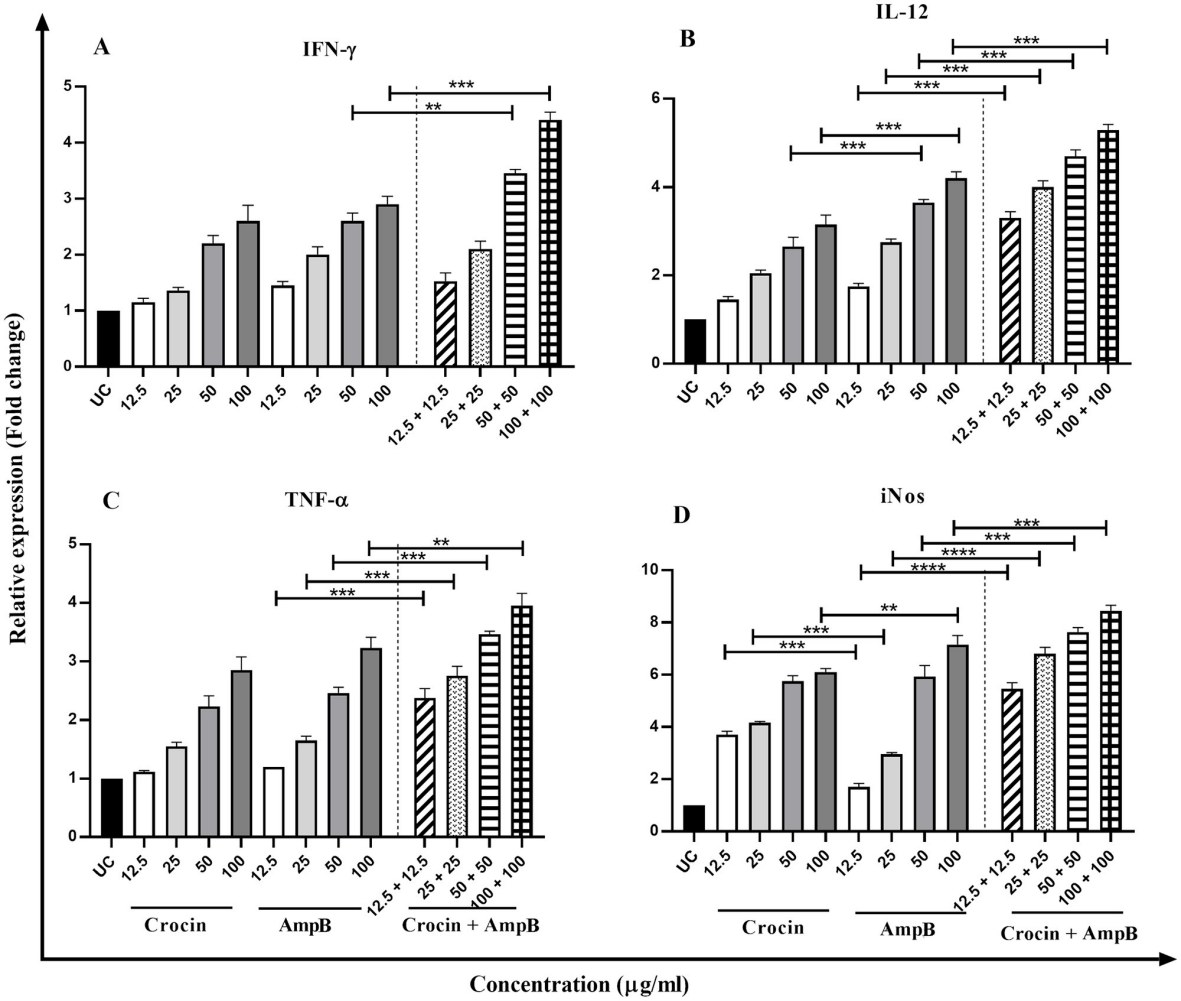
Th1 cytokines expression profile of IFN-γ (A), IL-12p40 (B), iNOS (C), and TNF-α (D) in macrophages treated with different concentrations of crocin, amphotericin B (AmpB), and combination compared to the untreated control group. Error bars are SD (**P < 0.01, ***P < 0.001, and ****P < 0.0001). Each test was performed thrice.

**Fig 7:**
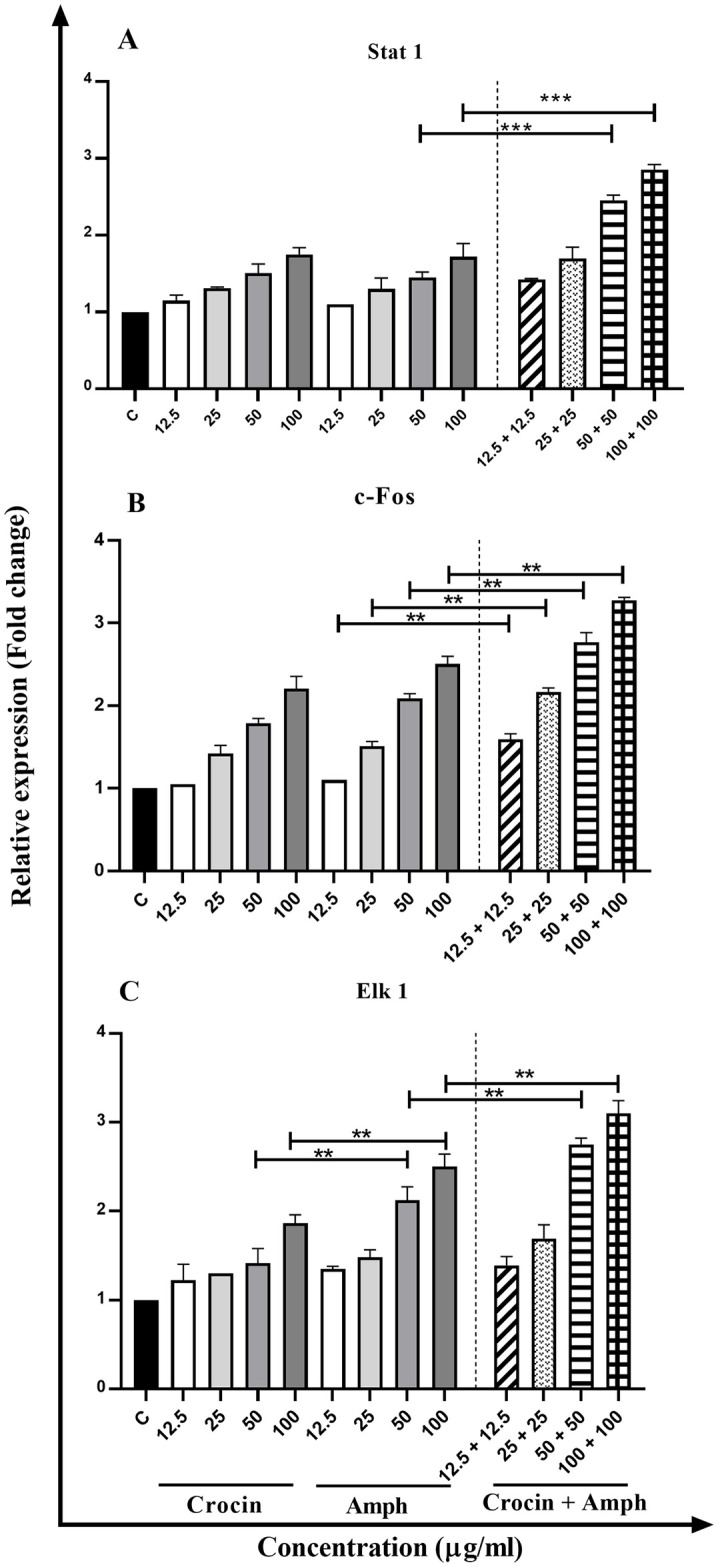
Transcription factors expression profile of STAT1 (A), c-Fos (B), and Elk-1 (C) in macrophages treated with different concentrations of crocin, amphotericin B (AmpB), and crocin plus AmpB compared to the untreated control group. Error bars are SD (*P < 0.05, **P < 0.01, and ***P < 0.001). Each test was performed thrice.

**Fig 8:**
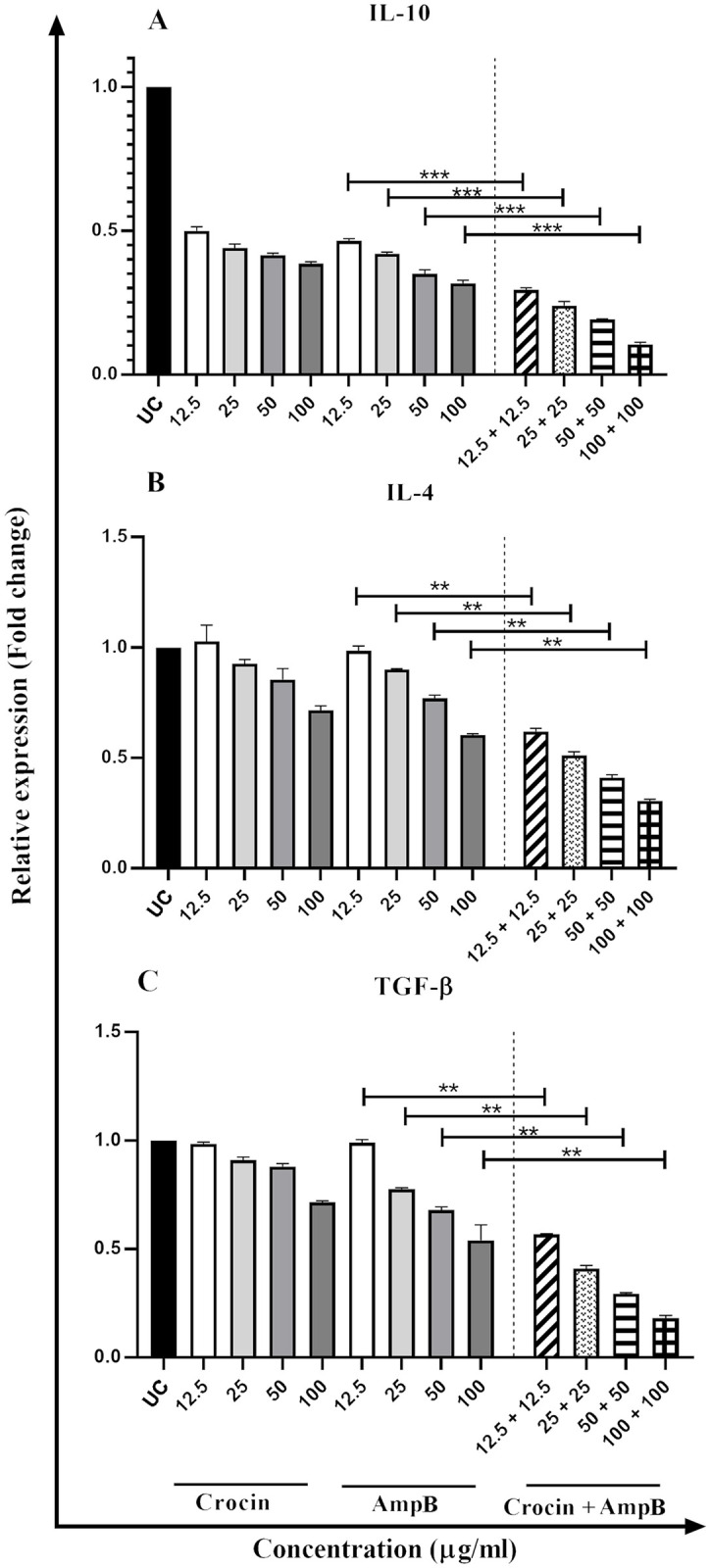
Th2 cytokines expression profile of IL-10 (A), IL-4 (B), and TGF-β (C) in macrophages treated with different concentrations of crocin, amphotericin B (AmpB), and crocin plus AmpB compared to the untreated control group. Error bars are SD (**P < 0.01, ***P < 0.001, and **** *P* < 0.0001). Each test was performed thrice.

Comparison of cytokines expression of Th1 cytokines (IFN-γ, IL-12p40, and TNF-α) (Fig 6), iNOS, and STAT1, c-Fos, and Elk-1 (Fig 7) displayed elevated levels in treated macrophages compared untreated control group. On the other hand, IL-4, IL-10, and TGF-β gene expression (as a marker of the Th2 pathway) were reduced by increasing drug concentrations (Fig 8). The gene expression profiles in Th1 and Th2 phenotypes and transcription factors in the crocin and AmpB alone were the same. However, a significant upsurge of Th1 line and transcription genes and a substantial diminution in the Th2 subset at comparable concentrations were detected (*P* < 0.001).

### Effect of crocin, AmpB, and combination on NO generation

**Fig 9:**
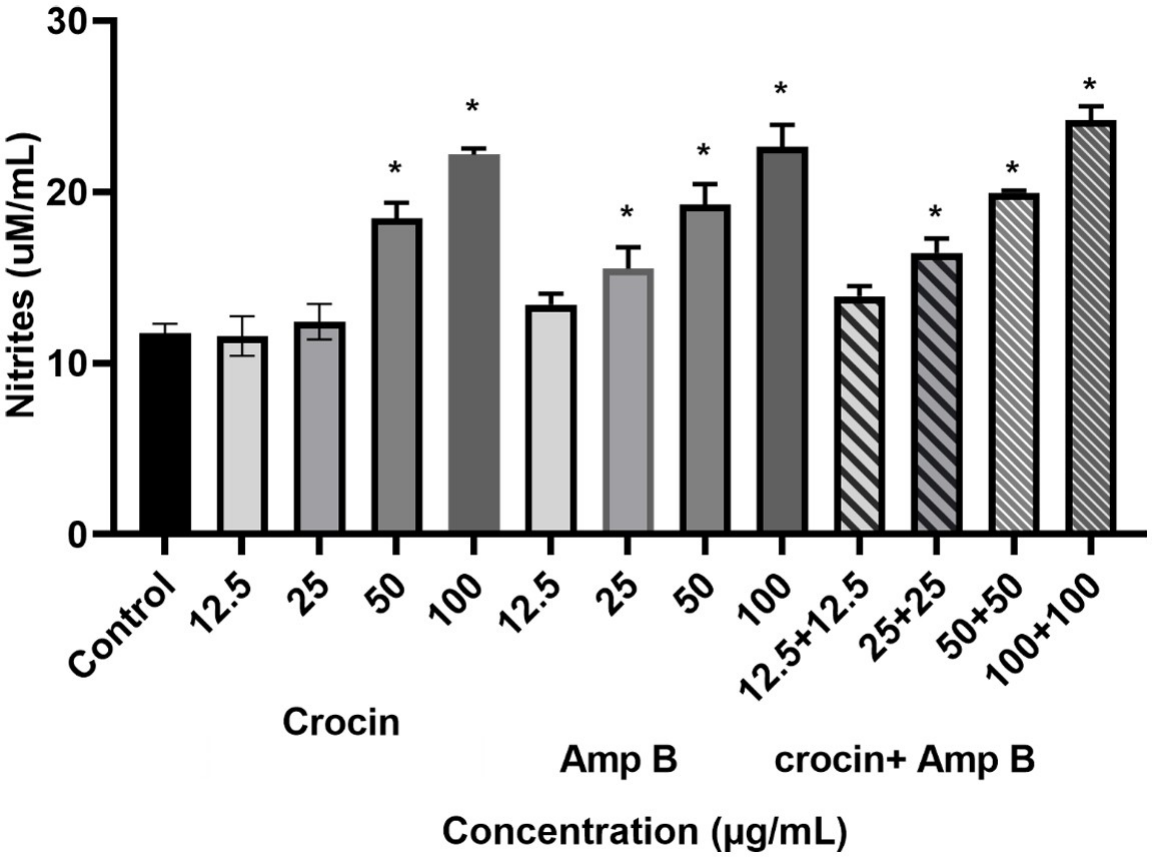
Effect of crocin, amphotericin B (AmpB), and combination on nitrite generation in macrophages infected by *Leishmania*. *major* compared to the untreated control (* *P* < 0.001).

The results presented that crocin and AmpB alone or in combination could significantly increase nitrite production in macrophages infected by *L*. *major* by increasing drug concentrations relative to the untreated control group (Fig 9). While at lower concentrations of crocin (12.5 μM and 25 μM), AmpB (12.5 μM or combination (12.5+12.5 μM), no effect was observed.

### Effect of crocin, AmpB, and combination on ROS production

**Fig 10:**
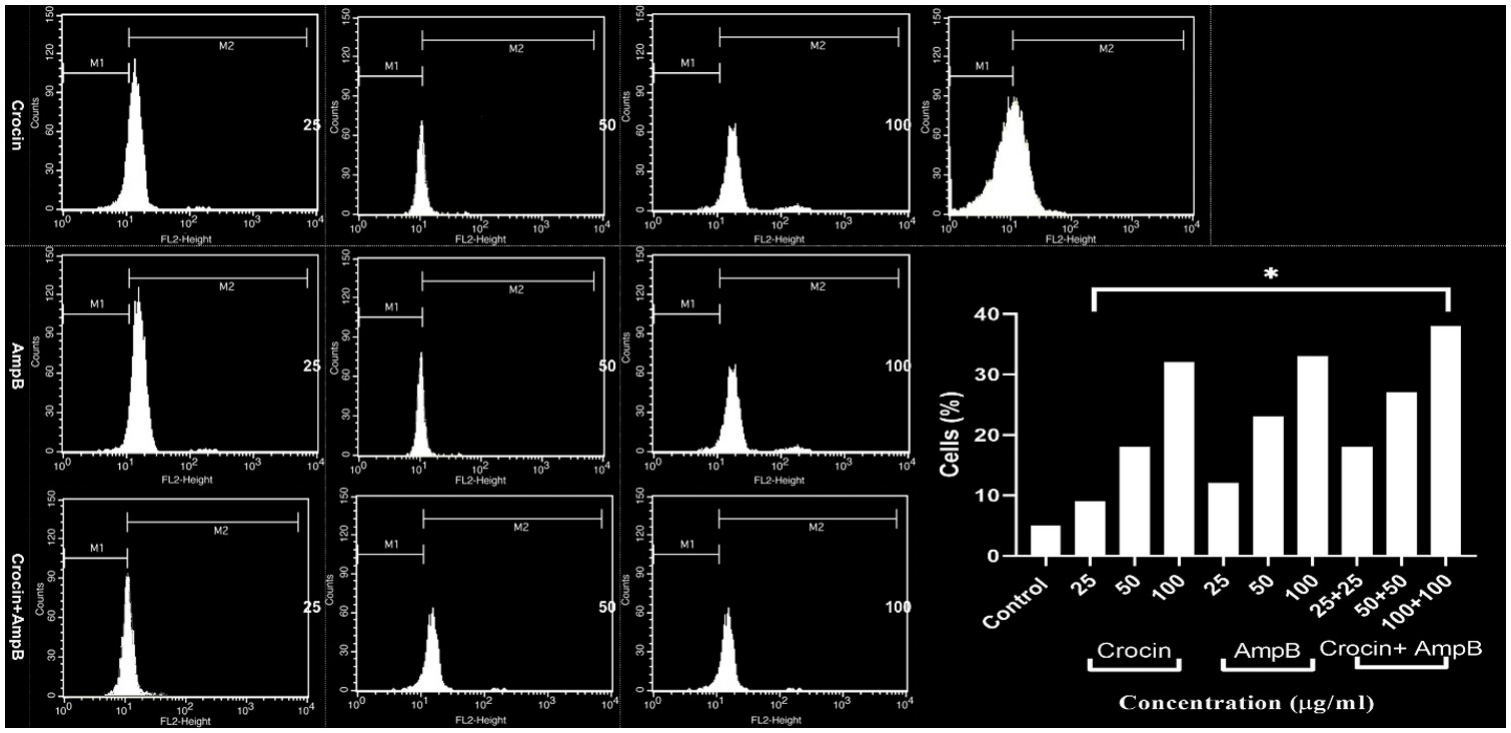
Effect of crocin, amphotericin B (AmpB), and combination on ROS in each treatment intra-macrophage *Leishmania*. *major* amastigotes (*P < 0.001 compared to the untreated control group).

Crocin, AmpB alone, or in combination similarly promoted the ROS level (*P* < 0.001) in the treated intra-macrophage amastigotes following a dose-response profile compared to untreated control (Fig 10).

### Effect of crocin, AmpB, and combination on cell cycle of *L*. *major* promastigotes

**Fig 11:**
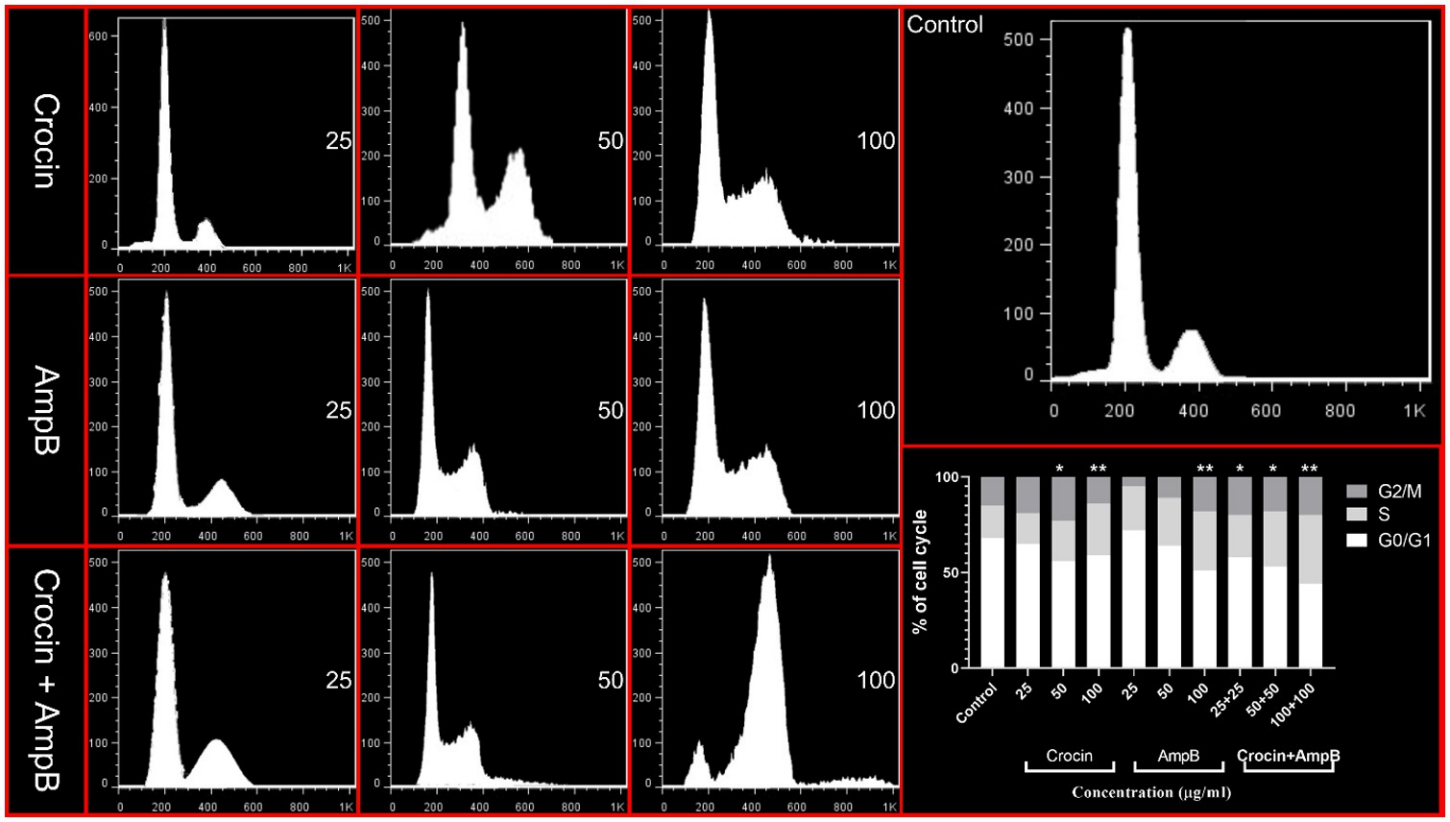
Effect of crocin, amphotericin B (AmpB), and combination on cycle arrest of *Leishmania major* promastigotes. (*P < 0.05 and **P < 0.001 compared to the untreated control group).

Crocin at 50 μM (*P* < 0.05) and 100 μM (*P* < 0.001), AmpB at 100 μM (*P* < 0.001) alone, and in combination at 50 μM+50 μM (*P* < 0.05) and 100 μM+100 μM (*P* < 0.001) significantly triggered induction at the sub-G0/G1 phase (Fig 11).

### Effect of crocin, AmpB, and combination on programmed cell death in *L*. *major*

**Fig 12:**
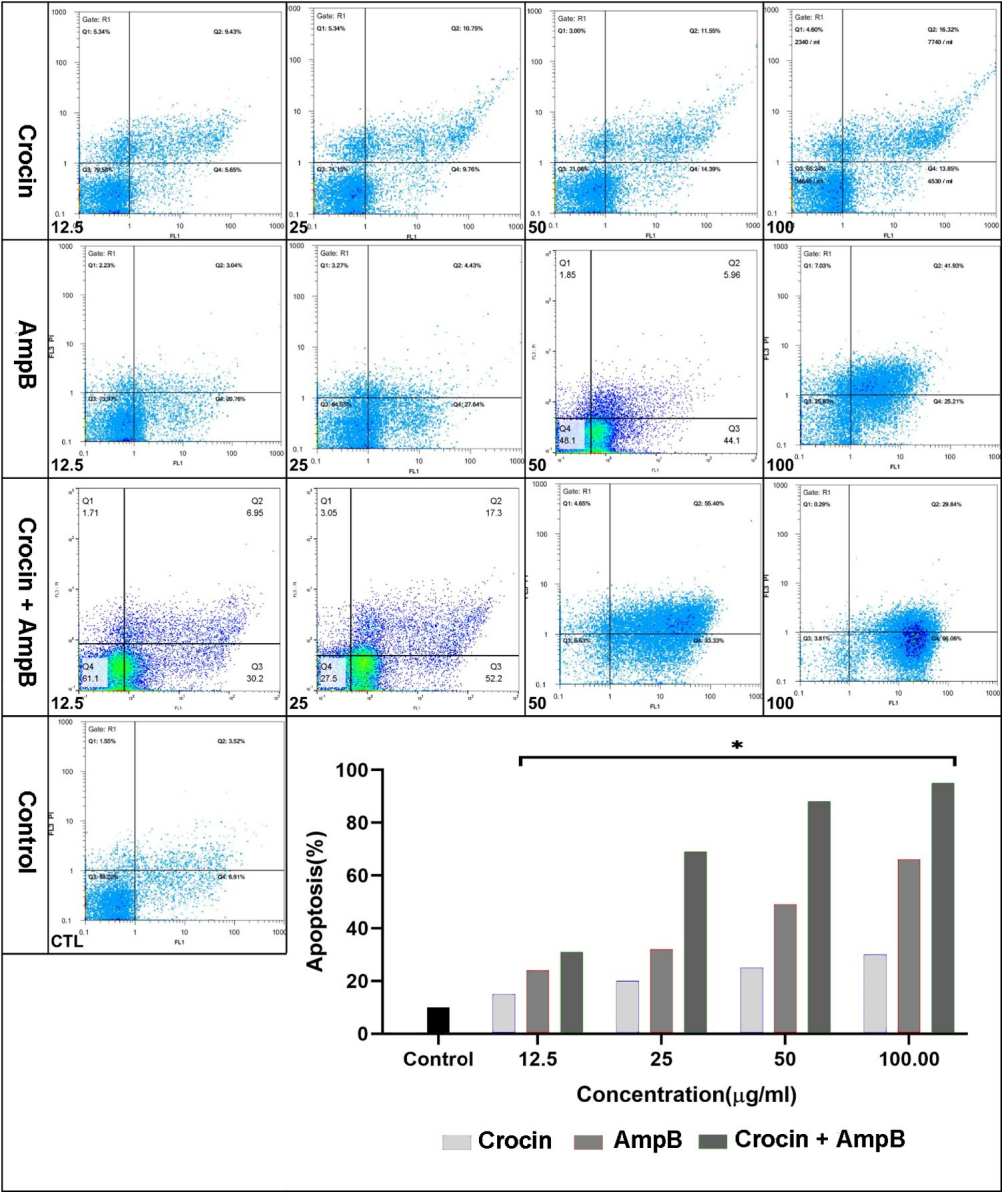
Effect of crocin, amphotericin B (AmpB), and combination on programmed cell death of *Leishmania major* promastigotes. (*P < 0.001 compared to the untreated control group, **P < 0.001 combination treatment group compared to the AmpB group).

Different concentrations of crocin, AmpB, or combination significantly induced the apoptotic profiles compare untreated control group (*P* < 0.001), except crocin alone at 12.5 μM showed no effect. In combination therapy, the PCD significantly increased compared to AmpB or crocin treated alone (Fig 12).

## Discussion

Existing drugs used to treat leishmaniasis include antimonials, AmpB, miltefosine, allopurinol, pentamidine, and azole compounds [34]. However, these drugs are often ineffective, and expensive, associated with the emergence of drug resistance. The desperate need for alternative therapeutics or the co-administration of other therapeutics is crucial. Natural products and phytomedicines have represented a well-established and valued source of potentially bioactive compounds in drug discovery, resulting in a durable interest in developing natural products to combat all forms of leishmaniasis [35, 36]. This complex disease is endemic in low-income countries with little incentive for pharmaceutical companies to participate in developing new drugs. Without proper medical and health infrastructures, people in these areas increasingly appreciate natural medicines as therapeutics.

This study showed that crocin established an antileishmanial activity. However, the lethal effect was more significantly enhanced when combined with AmpB. The higher efficiency of the crocin and AmpB mixture in hindering the propagation of *L*. *major* in macrophage assays was mediated by elevating immune elements and preventing *Leishmania* arginase (L-ARG) levels. The *Leishmania* arginase activity has focused on intense investigations [36]. L-ARG is the primary enzyme in *Leishmania* polyamine biosynthesis. Recent studies have revealed the significance of polyamines for *Leishmania* persistence, growth, differentiation, and infectivity and corroborated this biochemical pathway as a critical therapeutic drug target [37]. The current results displayed that the cell treated with different crocin/AmpB mixture concentrations inhibited L-ARG uptake and enhanced the parasite killing. This finding is consistent with nicotinamide inhibition of L-ARG on *L*. *tropica*, the causative agent of urban CL [38].

Arginine is also the precursor in the biosynthesis of many proteins and nitric oxide (NO). Hence, arginine is crucial to produce NO in phagocytic cells through iNOS potentiation of the immune response, contributing to parasite death [39]. Crocin can trigger adaptive immune functions via T CD^+^4 lymphocytes associated-cytokine production, a prominent Th1 phenotype cell line polarization towards the significant immune expression of cytokines including TNF-α, IFN-ɣ, iNOS, and allied transfer factors (Stat-1, c-Fos, and Elk-1). Recovery from CL typically depends on introducing T-cell proliferation, predominantly Th1 response, primed by IL-12p40, dendritic cells (DCs), and macrophages [40]. IFN-ɣ produced from IL-12p40 signed T-lymphocytes stimulate tumor necrosis factor (TNF)-α and NO-mediated alleviation of the organisms [41]. Similar to the potentiation of IL-12p40 and supplementary pro-inflammatory cytokines, expression levels of Th2 cytokines have likewise been broadly explored [42]. IL-4, IL-10, and TGF-β prevent the production of IFN-γ released from macrophages. IL-4 is recognized to inhibit macrophage stimulation effectively, but IL-10 plays a pivotal role in CL evolution.

Modulating immune responses with natural remedies and secondary products has been confirmed as a promising therapeutic approach [43, 44]. Approximately 80% of the global public still uses herbal medicine for their clinical and healthcare requirements [38, 45]. World Health Organization (WHO) rationalized its Traditional Medicine Strategy for 2014–2023 to promote medicinal plants [46, 47]. The strategy aims to support the countries in developing proactive campaigns and reinforce traditional medicine’s role in keeping populations healthy. The immunostimulatory phytochemicals are the primary and rational basis of potential leishmanicidal and may provide new strategies to combat leishmaniasis, alone or as a combination. Many plant-extracted macromolecules revealed strong properties on immune system roles in experimental models and signified their beneficial potential [38, 48–50].

The chief active derivative of saffron is crocin [51]. Crocin, a saffron glycoside, is a carotenoid having four analogs: crocin 1, crocin 2, crocin 3, and crocin. Reports have indicated that saffron possessed practical value with numerous pharmacological properties, including antioxidant, anti-inflammatory, and cardioprotective effects. Some studies showed that crocin protects against many degenerative chronic diseases [52, 53]. A study revealed that the oral treatment of crocin within one month in healthy volunteers was safe compared with the control group [54].

In the life cycle, leishmania parasites infect mammalian macrophages where the internalization of organisms triggers substantial quantities of ROS mediates to control infection, as they are significantly expressed in this study. The mechanism of action of different antileishmanial active ingredients, including crocin/AmpB, involves the generation of ROS to enable the killing process of the organism. High volumes of ROS are fatal to promastigote and amastigote forms of *Leishmania donovani*. A study showed that *L*. *donovani* responsible promastigote stage undergoes PCD on treatment with H_2_O_2_ [55]. High levels of ROS are reported to be a deadly weapon exerted by phagocytic cells for impairment of critical cellular organic substances like proteins, lipids, and DNA, thus resulting in apoptosis of the causative agent [56, 57]. ROS in leishmanial agents could be induced due to cellular and drug uptake [58]. Various chemotherapeutic compounds used against *Leishmania* species or cancer treatment facilitate their effects by generating ROS [59–63]. The crucial docking technique was devised to anticipate drug-receptor interactions due to the strong crocin and NO affinity and the formation of large quantities of oxidative metabolites [27].

Considering the fundamental biology of *Leishmania* species requires thoughtful of the cell cycle. We have established that *L*. *major* undergoes significant changes in ultrastructural profile [64]. Different cell cycle phases may be seen in a broad spectrum of microstructural features found in cultures during exponential development. Current data displayed that treatment with crocin/AmpB perturbed the *Leishmania* cell cycle growth, or mitosis, and promoted DNA synthesis. Induced cell propagation arrest utilizes natural components to stop progression through the cell cycle. This feature could be an indicator for evaluating and monitoring the experimental drugs’ action.

The leishmanicidal effect of crocin/AmpB was also mediated through apoptotic-like effects as evidenced by phosphatidylserine (PS) externalization [49, 65]. This significant change appears due to reduced phospholipids translocase activity and activation of a calcium-dependent scramblase. Exposure of PS on the exterior membrane of the cells is an apparent change common to various apoptotic cells and cell-cycle arrest at different stages of the growth phase. Generally, these findings indicate that crocin/AmpB has a promising antileishmanial effect facilitated by programmed cell death and further advanced study as a possible therapeutic choice for the treatment of leishmaniasis.

Antimonials have been changed with AmpB per the instructions for treating leishmaniasis. AmpB is the second-line treatment for antimony strains that have developed resistance [66]. This polyene antifungal drug attaches to ergosterol to target the cell walls of promastigotes and amastigotes [67, 68]. Leishmaniasis is one of several diseases for which AmpB has been employed for decades as an authorized medicine. AmpB might work in concert with paromomycin or miltefosine to reduce the extracellular promastigotes, suppress intracellular amastigotes, and restrict the disease over time [69]. Combining plant immunomodulators with traditional medications like AmpB may enable the effective treatment of a variety of molecular targets, improving therapeutic effectiveness and reducing toxicity [70].

Liposomal AmpB has been used extensively to treat VL due to its higher safety and efficacy profile [71]. It is the standard drug for immunocompetent patients, and the combination treatment is an acceptable choice because of the likely chemical achievement of two medicines in lower dosages with different mechanistic actions. The rationale behind combination or polytherapy is to increase activity by using compounds with synergistic or additive interaction. This approach has increasingly been supported to upsurge treatment efficacy, reduce treatment period/cost, and delay or halt the emergence of recrudescence and resistance [72]. Based on crocin’s antileishmanial effect and targeting to detect potential new therapeutic alternatives for leishmaniasis treatment, this study was accomplished to evaluate the activity of crocin in combination with AmpB as a conventional antileishmanial drug. The outcomes are very encouraging and may further support the justification of combination therapy for CL to provide safe and effective options.

In conclusion, this study presented the highest effect of the crocin/AmpB combination in hindering the multiplication of *L*. *major* stages in a macrophage assay by inhibiting L-ARG levels, potentiating immune response, and arresting cell cycle growth. Therefore, with multiple mechanistic actions, a high safety index on mammalian cells, and potent antioxidative activity combined with AmpB, crocin could be esteemed as a basis for a potential bioactive component and a logical source for leishmanicidal drug development against CL in future advanced clinical settings.

## Supporting information

S1 Dataset Data of analysis by one way ANOVA test for promastigote mortality, arginase activity, gene expression, NO generation, ROS production, cell cycle and program cell death. (ZIP)

## References

[1] Volpedo G, Huston RH, Holcomb EA, Pacheco-Fernandez T, Gannavaram S, Bhattacharya P, et al. From infection to vaccination: reviewing the global burden, history of vaccine development, and recurring challenges in global leishmaniasis protection. Expert Rev Vaccines. 2021; 1–16. doi: 10.1080/14760584.2021.1969231 34511000

[2] Tabasi M, Alesheikh AA, Sofizadeh A, Saeidian B, Pradhan B, AlAmri A. A spatio-temporal agent-based approach for modeling the spread of zoonotic cutaneous leishmaniasis in northeast Iran. Parasit Vectors. 2020;13: 1–17. 33176858 10.1186/s13071-020-04447-x PMC7659076

[3] WHO. Leishmaniasis. In: World Health Organization [Internet]. 2022. https://www.who.int/leishmaniasis/en/

[4] Torres-Guerrero E, Quintanilla-Cedillo MR, Ruiz-Esmenjaud J, Arenas R. Leishmaniasis: a review. F1000Research. 2017;6. doi: 10.12688/f1000research.11120.1 28649370 PMC5464238

[5] Ruiz-Postigo JA, Jain S, Mikhailov A, Maia-Elkhoury AN, Valadas S, Warusavithana S, et al. Global leishmaniasis surveillance: 2019–2020, a baseline for the 2030 roadmap/Surveillance mondiale de la leishmaniose: 2019–2020, une periode de reference pour la feuille de route a l’horizon 2030. Wkly Epidemiol Rec. 2021;96: 401–420.

[6] Knight CA, Harris DR, Alshammari SO, Gugssa A, Young T, Lee CM. Leishmaniasis: Recent epidemiological studies in the Middle East. Front Microbiol. 2023;13: 1052478. doi: 10.3389/fmicb.2022.1052478 36817103 PMC9932337

[7] Bogdan C. Macrophages as host, effector and immunoregulatory cells in leishmaniasis: impact of tissue micro-environment and metabolism. Cytokine X. 2020; 100041. doi: 10.1016/j.cytox.2020.100041 33604563 PMC7885870

[8] Carvalho AM, Guimarães LH, Costa R, Saldanha MG, Prates I, Carvalho LP, et al. Impaired Th1 Response Is Associated With Therapeutic Failure in Patients With Cutaneous Leishmaniasis Caused by Leishmania braziliensis. J Infect Dis. 2021;223: 527–535. doi: 10.1093/infdis/jiaa374 32620011 PMC7881333

[9] Jafarzadeh A, Jafarzadeh S, Sharifi I, Aminizadeh N, Nozari P, Nemati M. The importance of T cell-derived cytokines in post-kala-azar dermal leishmaniasis. Cytokine. 2021;147: 155321. doi: 10.1016/j.cyto.2020.155321 33039255

[10] Madusanka RK, Silva H, Karunaweera ND. Treatment of cutaneous leishmaniasis and insights into species-specific responses: a narrative review. Infect Dis Ther. 2022; 1–17. doi: 10.1007/s40121-022-00602-2 35192172 PMC8960542

[11] Salari S, Bamorovat M, Sharifi I, Almani PGN. Global distribution of treatment resistance gene markers for leishmaniasis. J Clin Lab Anal. 2022; e24599. doi: 10.1002/jcla.24599 35808933 PMC9396204

[12] Bahraminegad S, Pardakhty A, Sharifi I, Ranjbar M. Therapeutic effects of the as-synthesized polylactic acid/chitosan nanofibers decorated with amphotricin B for in vitro treatment of Leishmaniasis. J Saudi Chem Soc. 2021;25: 101362.

[13] Roatt BM, de Oliveira Cardoso JM, De Brito RCF, Coura-Vital W, de Oliveira Aguiar-Soares RD, Reis AB. Recent advances and new strategies on leishmaniasis treatment. Appl Microbiol Biotechnol. 2020; 1–13. doi: 10.1007/s00253-020-10856-w 32875362

[14] Passero LFD, Brunelli E dos S, Sauini T, Amorim Pavani TF, Jesus JA, Rodrigues E. The Potential of Traditional Knowledge to Develop Effective Medicines for the Treatment of Leishmaniasis. Front Pharmacol. 2021;12: 1408. doi: 10.3389/fphar.2021.690432 34220515 PMC8248671

[15] Cardone L, Castronuovo D, Perniola M, Cicco N, Candido V. Saffron (Crocus sativus L.), the king of spices: An overview. Sci Hortic (Amsterdam). 2020;272: 109560.

[16] Caser M, Demasi S, Stelluti S, Donno D, Scariot V. Crocus sativus L. Cultivation in alpine environments: Stigmas and tepals as source of Bioactive Compounds. Agronomy. 2020;10: 1473.

[17] Xue XH. Cultivation of Crocus sativus. Zhong yao tong bao (Beijing, China 1981). 1982;7: 3–4. 6215174

[18] Lambrianidou A, Koutsougianni F, Papapostolou I, Dimas K. Recent advances on the anticancer properties of saffron (Crocus sativus L.) and its major constituents. Molecules. 2021;26: 86. 10.3390/molecules26010086 PMC7794691 33375488

[19] Zeka K, Marrazzo P, Micucci M, Ruparelia KC, Arroo RRJ, Macchiarelli G, et al. Activity of Antioxidants from Crocus sativus L. Petals: Potential Preventive Effects towards Cardiovascular System. Antioxidants. 2020;9: 1102. doi: 10.3390/antiox9111102 33182461 PMC7697793

[20] Kumar V, Bhat ZA, Kumar D, Khan NA, Chashoo IA, Shah MY. Pharmacological profile of crocus sativus-a comprehe sive review. Pharmacologyonline. 2011;3: 799–811.

[21] Srivastava R, Ahmed H, Dixit RK. Crocus sativus L.: a comprehensive review. Pharmacogn Rev. 2010;4: 200. doi: 10.4103/0973-7847.70919 22228962 PMC3249922

[22] Zeka K, Ruparelia KC, Continenza MA, Stagos D, Vegliò F, Arroo RRJ. Petals of Crocus sativus L. as a potential source of the antioxidants crocin and kaempferol. Fitoterapia. 2015;107: 128–134. doi: 10.1016/j.fitote.2015.05.014 26012879

[23] Fernández-Albarral JA, De Hoz R, Ramírez AI, López-Cuenca I, Salobrar-García E, Pinazo-Durán MD, et al. Beneficial effects of saffron (Crocus sativus L.) in ocular pathologies, particularly neurodegenerative retinal diseases. Neural Regen Res. 2020;15: 1408. doi: 10.4103/1673-5374.274325 31997799 PMC7059587

[24] Morelle C, Mukherjee A, Zhang J, Fani F, Khandelwal A, Gingras H, et al. Well-Tolerated Amphotericin B Derivatives That Effectively Treat Visceral Leishmaniasis. ACS Infect Dis. 2021;7: 2472–2482. doi: 10.1021/acsinfecdis.1c00245 34282886

[25] Alves MM de M, Arcanjo DDR, Figueiredo KA, Oliveira JS de SM, Viana FJC, Coelho E de S, et al. Gallic and ellagic acids are promising adjuvants to conventional amphotericin B for the treatment of cutaneous leishmaniasis. Antimicrob Agents Chemother. 2020;64: e00807–20. doi: 10.1128/AAC.00807-20 32928735 PMC7674045

[26] Ranjbar R, Shayanfar P, Maniati M. In Vitro Antileishmanial Effects of Saffron Compounds, Crocin and Stigmasterol, on Iranian Strain of Leishmania major (MHOM/IR/75/ER). Iran J Parasitol. 2021;16: 151. doi: 10.18502/ijpa.v16i1.5535 33786057 PMC7988674

[27] Formaglio P, Alabdullah M, Siokis A, Handschuh J, Sauerland I, Fu Y, et al. Nitric oxide controls proliferation of Leishmania major by inhibiting the recruitment of permissive host cells. Immunity. 2021;54: 2724–2739. doi: 10.1016/j.immuni.2021.09.021 34687607 PMC8691385

[28] Sumbalova L, Stourac J, Martinek T, Bednar D, Damborsky J. HotSpot Wizard 3.0: web server for automated design of mutations and smart libraries based on sequence input information. Nucleic Acids Res. 2018;46: W356–W362. doi: 10.1093/nar/gky417 29796670 PMC6030891

[29] Tian W, Chen C, Lei X, Zhao J, Liang J. CASTp 3.0: computed atlas of surface topography of proteins. Nucleic Acids Res. 2018;46: W363–W367. doi: 10.1093/nar/gky473 29860391 PMC6031066

[30] Liu N, Song M, Wang N, Wang Y, Wang R, An X, et al. The effects of solid-state fermentation on the content, composition and in vitro antioxidant activity of flavonoids from dandelion. PLoS One. 2020;15: e0239076. doi: 10.1371/journal.pone.0239076 32931505 PMC7491732

[31] Bahraminejad S, Pardakhty A, Sharifi I, Ranjbar M, Karami-Mohajeri S, Sharifi F. Preparation and Evaluation of Physicochemical Properties and Anti-leishmanial Activity of Zirconium/Tioxolone Niosomes Against Leishmania major. Arab J Chem. 2022; 104156.

[32] Bahraminegad S, Pardakhty A, Sharifi I, Ranjbar M. The assessment of apoptosis, toxicity effects and anti-leishmanial study of Chitosan/CdO core-shell nanoparticles, eco-friendly synthesis and evaluation. Arab J Chem. 2021;14: 103085.

[33] Askari VR, Shafiee-Nick R. Promising neuroprotective effects of β-caryophyllene against LPS-induced oligodendrocyte toxicity: A mechanistic study. Biochem Pharmacol. 2019;159: 154–171. 30529211 10.1016/j.bcp.2018.12.001

[34] Burza S, Croft SL, Boelaert M. Leishmaniasis. Lancet. 2018;392: 951–970. doi: 10.1016/S0140-6736(18)31204-2 30126638

[35] Gervazoni LFO, Barcellos GB, Ferreira-Paes T, Almeida-Amaral EE. Use of natural products in leishmaniasis chemotherapy: an overview. Front Chem. 2020;8: 1031. doi: 10.3389/fchem.2020.579891 33330368 PMC7732490

[36] Carter NS, Stamper BD, Elbarbry F, Nguyen V, Lopez S, Kawasaki Y, et al. Natural Products That Target the Arginase in Leishmania Parasites Hold Therapeutic Promise. Microorganisms. 2021;9: 267. doi: 10.3390/microorganisms9020267 33525448 PMC7911663

[37] Malta-Santos H, França-Costa J, Macedo A, Queiroz ATL, Fukutani KF, Muxel SM, et al. Differential expression of polyamine biosynthetic pathways in skin lesions and in plasma reveals distinct profiles in diffuse cutaneous leishmaniasis. Sci Rep. 2020;10: 1–12. 32601369 10.1038/s41598-020-67432-5 PMC7324605

[38] Oliaee RT, Sharifi I, Bamorovat M, Keyhani A, Babaei Z, Salarkia E, et al. The potential role of nicotinamide on Leishmania tropica: An assessment of inhibitory effect, cytokines gene expression and arginase profiling. Int Immunopharmacol. 2020;86: 106704. doi: 10.1016/j.intimp.2020.106704 32590317

[39] Bodhale N, Ohms M, Ferreira C, Mesquita I, Mukherjee A, André S, et al. Cytokines and metabolic regulation: A framework of bidirectional influences affecting Leishmania infection. Cytokine. 2021;147: 155267. doi: 10.1016/j.cyto.2020.155267 32917471

[40] Ikeogu NM, Akaluka GN, Edechi CA, Salako ES, Onyilagha C, Barazandeh AF, et al. Leishmania immunity: advancing immunotherapy and vaccine development. Microorganisms. 2020;8: 1201. doi: 10.3390/microorganisms8081201 32784615 PMC7465679

[41] Dubie T, Mohammed Y. Review on the Role of Host Immune Response in Protection and Immunopathogenesis during Cutaneous Leishmaniasis Infection. J Immunol Res. 2020;2020. doi: 10.1155/2020/2496713 32656269 PMC7320295

[42] Soares-Silva M, Diniz FF, Gomes GN, Bahia D. The mitogen-activated protein kinase (MAPK) pathway: role in immune evasion by trypanosomatids. Front Microbiol. 2016;7: 183. doi: 10.3389/fmicb.2016.00183 26941717 PMC4764696

[43] Mahmoudvand H, Sharififar F, Rahmat S, Tavakoli R, Saedi Dezaki E, Jahanbakhsh S, et al. Evaluation of antileishmanial activity and cytotoxicity of the extracts of Berberis vulgaris and Nigella sativa against Leishmania tropica. J Vector Borne Dis. 2014;51. 25540961

[44] Mahmoudvand H, S Dezaki E, Ezatpour B, Sharifi I, Kheirandish F, Rashidipour M. In vitro and in vivo antileishmanial activities of Pistacia vera essential oil. Planta Med. 2016;82: 279–284. doi: 10.1055/s-0035-1558209 26829519

[45] Keshav P, Goyal DK, Kaur S. Antileishmanial potential of immunomodulator gallic acid against experimental murine visceral leishmaniasis. Parasite Immunol. 2021;43: e12875. doi: 10.1111/pim.12875 34347892

[46] Oryan A. Plant-derived compounds in treatment of leishmaniasis. Iran J Vet Res. 2015;16: 1. 27175144 PMC4789233

[47] Burton A, Smith M, Falkenberg T. Building WHO’s global strategy for traditional medicine. Eur J Integr Med. 2015;7: 13–15.

[48] Nair A, Chattopadhyay D, Saha B. Plant-derived immunomodulators. New Look to Phytomedicine. Elsevier; 2019. pp. 435–499.

[49] Keyhani A, Sharifi I, Salarkia E, Khosravi A, Oliaee RT, Babaei Z, et al. In vitro and in vivo therapeutic potentials of 6-gingerol in combination with amphotericin B for treatment of Leishmania major infection: Powerful synergistic and multifunctional effects. Int Immunopharmacol. 2021;101: 108274. doi: 10.1016/j.intimp.2021.108274 34688150

[50] Saduqi M, Sharifi I, Babaei Z, Keyhani A, Mostafavi M, Hakimi Parizi M, et al. Anti-leishmanial and immunomodulatory effects of epigallocatechin 3-o-gallate on leishmania tropica: Apoptosis and gene expression profiling. Iran J Parasitol. 2019;14. doi: 10.18502/ijpa.v14i4.2094 32099555 PMC7028242

[51] Rahaiee S, Moini S, Hashemi M, Shojaosadati SA. Evaluation of antioxidant activities of bioactive compounds and various extracts obtained from saffron (Crocus sativus L.): a review. J Food Sci Technol. 2015;52: 1881–1888. doi: 10.1007/s13197-013-1238-x 25829569 PMC4375186

[52] Alavizadeh SH, Hosseinzadeh H. Bioactivity assessment and toxicity of crocin: a comprehensive review. Food Chem Toxicol. 2014;64: 65–80. doi: 10.1016/j.fct.2013.11.016 24275090

[53] Mohamadpour AH, Ayati Z, Parizadeh M, Rajbai O, Hosseinzadeh H. Safety evaluation of crocin (a constituent of saffron) tablets in healthy volunteers. Iran J Basic Med Sci. 2013;16: 39. 23638291 PMC3637903

[54] Su X, Yuan C, Wang L, Chen R, Li X, Zhang Y, et al. The Beneficial Effects of Saffron Extract on Potential Oxidative Stress in Cardiovascular Diseases. Oxid Med Cell Longev. 2021;2021. doi: 10.1155/2021/6699821 33542784 PMC7840270

[55] Das M, Mukherjee SB, Shaha C. Hydrogen peroxide induces apoptosis-like death in Leishmania donovani promastigotes. J Cell Sci. 2001;114: 2461–2469. doi: 10.1242/jcs.114.13.2461 11559754

[56] Kiral F, Sekkin S, Pasa S, Ertabaklar H, Ulutas PA, Asici GSE. Investigation of DNA damage and protein damage caused by oxidative stress in canine visceral leishmaniasis. Med Weter Med Pract. 2021;77: 407–412.

[57] Roma EH, Macedo JP, Goes GR, Gonçalves JL, De Castro W, Cisalpino D, et al. Impact of reactive oxygen species (ROS) on the control of parasite loads and inflammation in Leishmania amazonensis infection. Parasit Vectors. 2016;9: 1–13. 27056545 10.1186/s13071-016-1472-y PMC4825088

[58] Adak S, Pal S. Ascorbate peroxidase acts as a novel determiner of redox homeostasis in Leishmania. Antioxid Redox Signal. 2013;19: 746–754. doi: 10.1089/ars.2012.4745 22703594

[59] Inacio JDF, Gervazoni L, Canto-Cavalheiro MM, Almeida-Amaral EE. The Effect of (-)-Epigallocatechin 3-O—Gallate In Vitro and In Vivo in Leishmania braziliensis: Involvement of Reactive Oxygen Species as a Mechanism of Action. PLoS Negl Trop Dis. 2014;8: e3093. doi: 10.1371/journal.pntd.0003093 25144225 PMC4140776

[60] Mehta A, Shaha C. Apoptotic death in Leishmania donovani promastigotes in response to respiratory chain inhibition: Complex II inhibition results in increased pentamidine cytotoxicity. J Biol Chem. 2004;279: 11798–11813. doi: 10.1074/jbc.M309341200 14679210

[61] Poltronieri J, B Becceneri A, M Fuzer A, Cesar Filho C, Martin CBM, Cezar Vieira P, et al. [6]-gingerol as a cancer chemopreventive agent: a review of its activity on different steps of the metastatic process. Mini Rev Med Chem. 2014;14: 313–321. doi: 10.2174/1389557514666140219095510 24552266

[62] Fonseca-Silva F, Inacio JDF, Canto-Cavalheiro MM, Almeida-Amaral EE. Reactive Oxygen Species Production and Mitochondrial Dysfunction Contribute to Quercetin Induced Death in Leishmania amazonensis. PLoS One. 2011;6: e14666. doi: 10.1371/journal.pone.0014666 21346801 PMC3035610

[63] Riezk A, Raynes JG, Yardley V, Murdan S, Croft SL. Activity of chitosan and its derivatives against Leishmania major and Leishmania mexicana in vitro. Antimicrob Agents Chemother. 2020;64: e01772–19. doi: 10.1128/AAC.01772-19 31871082 PMC7038302

[64] Wheeler RJ, Gluenz E, Gull K. The cell cycle of Leishmania: morphogenetic events and their implications for parasite biology. Mol Microbiol. 2011;79: 647–662. doi: 10.1111/j.1365-2958.2010.07479.x 21255109 PMC3166656

[65] Ilaghi M, Sharifi I, Sharififar F, Sharifi F, Oliaee RT, Babaei Z, et al. The potential role and apoptotic profile of three medicinal plant extracts on Leishmania tropica by MTT assay, macrophage model and flow cytometry analysis. Parasite Epidemiol Control. 2021;12: e00201. doi: 10.1016/j.parepi.2021.e00201 33511293 PMC7817489

[66] Firooz A, Mortazavi H, Khamesipour A, Ghiasi M, Abedini R, Balighi K, et al. Old world cutaneous leishmaniasis in Iran: clinical variants and treatments. J Dermatolog Treat. 2020; 1–11. doi: 10.1080/09546634.2019.1704214 31869258

[67] Stone NRH, Bicanic T, Salim R, Hope W. Liposomal amphotericin B (AmBisome^®^): a review of the pharmacokinetics, pharmacodynamics, clinical experience and future directions. Drugs. 2016;76: 485–500. 26818726 10.1007/s40265-016-0538-7 PMC4856207

[68] Mosimann V, Neumayr A, Paris DH, Blum J. Liposomal amphotericin B treatment of Old World cutaneous and mucosal leishmaniasis: a literature review. Acta Trop. 2018;182: 246–250. doi: 10.1016/j.actatropica.2018.03.016 29550282

[69] Hendrickx S, Van den Kerkhof M, Mabille D, Cos P, Delputte P, Maes L, et al. Combined treatment of miltefosine and paromomycin delays the onset of experimental drug resistance in Leishmania infantum. PLoS Negl Trop Dis. 2017;11: e0005620. doi: 10.1371/journal.pntd.0005620 28505185 PMC5444850

[70] Chouhan G, Islamuddin M, Sahal D, Afrin F. Exploring the role of medicinal plant-based immunomodulators for effective therapy of leishmaniasis. Front Immunol. 2014;5: 193. doi: 10.3389/fimmu.2014.00193 24829566 PMC4017133

[71] Khosravi A, Sharifi I, Tavakkoli H, Molaakbari E, Bahraminegad S, Salarkia E, et al. Cytotoxicity of Amphotericin B and AmBisome: In Silico and In Vivo Evaluation Employing the Chick Embryo Model. Front Pharmacol. 2022;13: 860598. doi: 10.3389/fphar.2022.860598 35754489 PMC9214246

[72] Goswami RP, Rahman M, Das S, Tripathi SK, Goswami RP. Combination therapy against Indian visceral Leishmaniasis with Liposomal Amphotericin B (FungisomeTM) and short-course miltefosine in comparison to miltefosine monotherapy. Am J Trop Med Hyg. 2020;103: 308. doi: 10.4269/ajtmh.19-0931 32394874 PMC7356435

